# Individual differences and the characterization of animal models of psychopathology: a strong challenge and a good opportunity

**DOI:** 10.3389/fphar.2013.00137

**Published:** 2013-11-08

**Authors:** Antonio Armario, Roser Nadal

**Affiliations:** ^1^Institut de Neurociències, Universitat Autònoma de BarcelonaBellaterra, Barcelona, Spain; ^2^Unitat de Fisiologia Animal, Facultat de Biociències, Universitat Autònoma de BarcelonaBellaterra, Barcelona, Spain; ^3^Unitat de Psicobiologia, Facultat de Psicologia, Universitat Autònoma de BarcelonaBellaterra, Barcelona, Spain

**Keywords:** individual differences, sex, anxiety, depression, stress, forced swim test, genetic selection, inbred strains

## Abstract

Despite the development of valuable new techniques (i.e., genetics, neuroimage) for the study of the neurobiological substrate of psychiatric diseases, there are strong limitations in the information that can be gathered from human studies. It is thus critical to develop appropriate animal models of psychiatric diseases to characterize their putative biological bases and the development of new therapeutic strategies. The present review tries to offer a general perspective and several examples of how individual differences in animals can contribute to explain differential susceptibility to develop behavioral alterations, but also emphasizes methodological problems that can lead to inappropriate or over-simplistic interpretations. A critical analysis of the approaches currently used could contribute to obtain more reliable data and allow taking full advantage of new and sophisticated technologies. The discussion is mainly focused on anxiety-like and to a lower extent on depression-like behavior in rodents.

## INTRODUCTION

It is now well-accepted that the development of pathologies in humans is related to individual differences determining either vulnerability or resilience. Therefore, individual differences in animal models of any disease are expected. If such differences are actually observed, these could allow us to further characterize factors contributing to them. On the other hand, if we suspect that a factor is involved in differential susceptibility to develop certain pathology, we can evaluate the predictive value of this factor. When searching for the differential susceptibility of animals to develop a certain behavioral disorder akin to a psychiatric disease we may be interested in specific aspects that include: (i)a more in depth behavioral characterization, (ii) the identification of biological markers, or (iii) the general goal of establishing neurobiological correlates of such a disease. In parallel we can study the putative genes involved. Unfortunately, numerous genes are usually involved in the development of most psychiatric diseases, with a minor contribution of each particular gene, making the study of the influence of particular genes extremely challenging. We will focus here on the impact of individual differences in putative animal models of psychiatric disorders, particularly anxiety, although some complementary references will be made to depression.

If we are interested in anxiety we need to identify tests to evaluate anxiety-like behavior (ALB) and models to induce hyper-anxiety in animals. An excellent overview of the genetic basis of ALB and some interpretative problems can be consulted (Clément et al., 2002). There are different tests advocated to evaluate ALB in rodents. At this point it is important to note that it is now well-accepted that we can distinguish between fear and anxiety (Davis et al., 2010). Fear appears in response to the actual presence of a precise danger (i.e., predator) or signals that precisely announce the appearance of danger (i.e., a sound signaling the imminence of a shock). In contrast, anxiety is generated in situations involving conflict or exposure to dangers that are not clearly signaled and have a high degree of unpredictability regarding the time and probability of appearance (i.e., the feeling of danger when rodents are exposed to a novel open space). Several tests have been proposed to evaluate anxiety and anxiolytics (Crawley, 1985; Treit, 1985). Some of them use the spontaneous behavior of animals in certain conditions, and others are based on classical or instrumental conditioned responses. The formers include activity/exploration in unknown environments of different configurations [open-field, hole-board, elevated plus-maze (EPM), or dark–light (D–L) boxes] or social interaction in unknown environments. Another unconditioned response, the acoustic startle response (ASR), has also been used as a marker of anxiety. The second group includes the conditioned burying test and several operant tasks such as active avoidance/escape responses, conditioned suppression, and Geller–Seifter conflict test. The overall impression is that there are no obvious advantages for the second as compared to the former tasks regarding evaluation of anxiolytics. Consequently, we will mainly refer to the former tasks, with special emphasis on the EPM, the most widely used in rodents, and, to a lesser extent, to the light–dark (L–D; or D–L) test and the ASR. The EPM consists of a plus-maze elevated over the floor, with two (closed) arms surrounded by walls and two others unprotected (open). The L–D apparatus has two compartments, one small and dark and another much greater and illuminated. In the L–D version the animals are initially put into the illuminated area and we measure the latency to enter for the first time in the dark compartment, the number of transitions between light and dark and the time spent in each compartment. In the D–L version, the animals are introduced into the dark compartment and the latency to enter into the illuminated area together with the other described measures are recorded. The EPM and L–D/D–L tests are based on the anxiety elicited in rodents (which are nocturnal animals) by open and illuminated spaces, and the natural tendency of these animals to explore new environments. These two opposite tendencies generated a conflict and we expect less emotional or less anxious animals to spend more time in the open arms of the EPM and the illuminated area of the L–D test.

There are two critical and interrelated questions behind the use of animal models in general that can apply to anxiety. The first is whether the test measures something related to anxiety in humans. The second is whether any individual behavioral test or particular measure within a test is capturing the essence of anxiety. It is generally assumed that anxiety, as other psychological traits, is likely to be a complex theoretical construct, and therefore it is naïve to assume that we can catch the essence of the trait with only one single test or one single measure. In fact, psychometric tests in humans assuming such a complexity, very often contain subscales measuring different components of anxiety. In contrast, most reports classifying animals in groups with putatively different levels of anxiety (or any other behavioral trait) are based on a single test and a single measure within a particular test (i.e., time spent in the open arms of the EPM) and this constitutes a strong limitation of these types of studies. The situation is even worse when we use the same approach to obtain genetically segregated lines/strains. Therefore, the approaches to this problem are in part conceptually different in animal models and humans and this can detract validity to animal studies.

There is another possible reason for the discrepancies among the tests that not necessarily relies on the fact that they measured different aspects of anxiety. Performance in a particular test may be influenced by factors (genes) other than anxiety that can perturb the actual relationship between the variable measured and the anxiety trait. The perturbing effects of those factors may be markedly different among lines/strains because of the random selection of genes related to these interfering factors. In this regard, Trullas and Skolnick (1993) in a seminal paper compared the magnitude of the ASR and the time spent in the open arm of the EPM in seven different mouse strains and showed the expected negative correlation between both variables when the average of values for each strain were included in the analysis. However, and importantly, when comparing only two particular strains, the relationship between the both parameter was erratic, likely because of the erratic contribution of random selection of non-anxiety-related genes to the performance of the animals in the two particular tests.

Exploratory factorial analysis has been repeatedly used to compare information given by different tests and to identify a putative anxiety factor. This analysis allows studying how different individual measures within a test or across different tests load on some statistically defined orthogonal (independent) axes. Nevertheless, it is important to note that those axes are in principle statistical not biological and we subjectively named these in function of the measures strongly loading on these factors. Using this approach, File et al. (1993) observed that time spent and number of entries in the open arms of the EPM strongly loaded on one factor that they called *anxiety* whereas number of entries in the closed arms loaded on another factor called *activity*. When factorial analysis has been applied to behavior in different tests presumably measuring anxiety, it is generally observed that key anxiety-related variables load on different factors, supporting the multi-factorial nature of anxiety (i.e., Belzung and Le Pape, 1994; Ramos and Mormède, 1998; Aguilar et al., 2002). It is thus not surprising that animals can differ in some tests of anxiety but not in others when for instance comparing strains or the consequences of certain genetic or environmental (i.e., acute or chronic stress) manipulations.

Factorial analysis allows not only identifying putative underlying factors but also evaluating the contribution of each factor to the variability observed in the population under study. As any particular behavioral variable is likely to be at least partially influenced by different underlying factors, it is possible that the contribution of each factor may change among different groups of animals. To illustrate this problem, factorial analysis of the behavior of male and female rats in different tests revealed a greater overall contribution of the factor *activity* in females and a greater contribution of the factor *anxiety* in males (Fernandes et al., 1999). It is expected this would happen when comparing strains or genetic or environmental manipulations and could contribute to explain quantitative differences in the loading of certain particular variables among different reports. It is particularly notable the discrepancies as to whether or not some variables related to activity load on the same factor as more anxiety-related measures (Ramos et al., 1997; Carola et al., 2002; Kanari et al., 2005; Milner and Crabbe, 2008; Takahashi et al., 2008). However, this is not so surprising. If we are working with high emotional animals, a marked reduction of any type of activity in novel environments is expected (also depending on the configuration of the environment), whereas such a generalized reduction of activity would be lower and more focused on the most dangerous parts of the apparatus in less emotional animals. In sum, exploratory factorial analysis can help to identify key behavioral measures associated with a particular trait, but we have to be aware that the quantitative relationship of these measures with the trait of interest can be modulated by a wide range of environmental and genetic factors. This can explain the discrepancies among the studies regarding the load of a particular measure on the factor of interest (i.e., anxiety), but also the inconsistencies in the relationship between key behavioral measures and biological parameters.

There are different, usually complementary, approaches to the study of individual differences that are summarized in **Table 1**: (a) the study of phenotypic differences in a non-selected outbred population of animals; (b) the study of animals genetically selected for a particular characteristic; (c) comparison of already available inbred strains; and (d) the study of genetically manipulated, mutant, animals.

**Table 1:** Different approaches for the characterization of individual differences.

Approach		Groups
Normal population (outbred)	Natural variations in trait A	Animals with high, intermediate, or low levels of the trait A
	Induced variations in trait A	Affected and non-affected animals, which have or not the trait A
	Polymorphisms of genes of interest	Animals with different alleles
Genetic selection for a trait (A or another)		Animals (inbred or outbred) with high or low levels of the trait (A or another)
Comparison of already existing inbred animals		Animals with different levels of the trait A
Genetic manipulation of targeted genes		Wild-type, heterozygous, or homozygous animals, with different levels of the trait A

*
After the selection or classification, several behavioral or biological measures are taken in the different groups of animals.
*

## INDIVIDUAL DIFFERENCES IN NON-SELECTED POPULATIONS

Depending on the main objectives, there are three main possibilities for the study of individual differences in non-genetically selected populations of animals (**Table 1**). If we suspect that anxiety could be associated to certain behavioral or biological characteristics, we can classify the animals on the basis of ALB and then study putative differences in those characteristics presumably related to this trait. If we are interested in how certain individual characteristics are predictive of the consequences of exposure to conditions, usually stress, that can elicit hyper-anxiety or depression, we classify the animals on the basis of the trait of interest and then expose the animals to environmental challenges. In this way we can support or reject the notion that there are differences in the consequences of stress that are related to the selected pre-stress trait. Finally, we can analyze how allelic variability in a population (polymorphisms) is related to the development of ALB.

### SEARCHING FOR ASSOCIATION BETWEEN A PARTICULAR TRAIT AND OTHER BEHAVIORAL OR BIOLOGICAL CHARACTERISTICS

We can first expose the animals to a particular test (i.e., the EPM), select a particular variable measured in the test (i.e., time spent in the open arms), and classify the animals by the median of the time spent in the open arms in low or high anxiety (LA or HA). In some cases, we could be interested in comparing only the extremes of the population after grouping it for instance in thirds or quartiles. After that, we would test the animals in other situations reflecting the traits we want to relate to anxiety. In addition to the problem already discussed of relying in one single measure, the above approaches require behavior of animals in the chosen test having a high degree of consistency when animals are repeatedly tested. One serious drawback is the fact that animal behavior can change after repeated exposure to the same situation so that the factors mainly contributing to a particular behavior can change. This is the case of the EPM and the forced swim test (FST).

The effects of prior experience with the EPM are not consistent across reports, some authors reporting changes and others not. Nevertheless, it has been repeatedly reported that experience of animals with the EPM can blunt the response to anxiolytics (i.e., File et al., 1993; Treit et al., 1993). Two different explanations have been offered: (a) during the first exposure animals would develop phobia to the open arms that is not sensitive to classical anxiolytics (File et al., 1993); or (b) during the first exposure, the activity is motivated by curiosity but also by searching for an escape (Roy et al., 2009). If the latter factors are reduced during the second exposure because the animals already knows that no escape is possible, the interest for exploring the more dangerous open arms can diminish and consequently drug-induced reduction of anxiety is not enough to overcome the low motivation to explore. Regardless of the final explanation, factorial analysis has confirmed that open arm entries are influenced by different factors during the first and the second exposure to the EPM (File et al., 1993; Holmes and Rodgers, 1998). In assessing test–retest reliability of the EPM measures results are also inconsistent, with good, intermediate, or bad reliabilities (i.e., Pellow et al., 1985; Lister, 1987; Andreatini and Bacellar, 2000). It is possible that discrepancies regarding the influence of prior experience with the EPM are related to the contextual memory capabilities to remember the novel environment during a second exposure as well as to the different contribution of anxiety versus motivation to explore among individuals and strains. Animals with poor contextual memory would be expected to perform more similarly during two exposures to the EPM. Animals in which a high motivation to explore predominates over or compensates for high levels of anxiety, may markedly reduce the open arms exploration when the environment is already known. On the contrary, animals in which HA predominates over motivation to explore during the first exposure may explore more the open arms during a second exposure when anxiety was probably reduced. In those animals whose contributing factors, whatever the reasons, do not markedly change from the test to the retest, a good correlation could be found, whereas this correlation would be low if contributing factors drastically change.

The FST was described by Porsolt et al. (1977) as a test to evaluate antidepressants. The classical procedure involves a pre-exposure (pre-test) to the water tank for 15 min, followed by administration of three doses of antidepressants over the next 24 h, the last administration 1h before a second exposure to the tank for 5 min. Time spent immobile during the test was the critical measure. We further introduced measurement of three different behaviors (struggling or climbing, mild swim, and immobility; Armario et al., 1988), an approach presently followed by most researchers. Moreover, we demonstrated that a pre-test was not needed to detect antidepressant activity in rats, although the efficacy of antidepressants was better after previous experience with the situation (Armario et al., 1988; Martí and Armario, 1993). It is well-known that forced swim behavior of animals can markedly change from the first to the second exposure (Porsolt et al., 1978). Changes in behavior from the pre-test to the test are likely to be the result of at least two opposite factors: the coping style (proactive or passive) of the animals and the learned experience about the inescapability of the situation. It is thus hardly surprising that not only the behavior during the first exposure but also the changes from the first to the second exposure are markedly different among strains of rats (Martí and Armario, 1996). In view of all these results it is considered that the FST can evaluate individual differences in coping strategies, certain environmental conditions (i.e., exposure to uncontrollable stress) enhancing passive (depression-like) coping that is reversed by antidepressants. Unfortunately, the relationship of the FST with other behavioral traits, particularly those also involving coping strategies, has been poorly explored in non-genetically selected populations. It is noteworthy in this regard that the FST has been scarcely compared with the tail suspension test (TST), another test based on the same principle as the FST that can only be applied to mice (Stéru et al., 1985). When such a comparison has been made using two different outbred mice strains, NMRI mice showed more immobility than CD1 mice in the TST, but the opposite was found in the FST (Vaugeois et al., 1997). Although we can hypothesize that the discrepancies are due to the contribution of specific factors to each test in addition to the common coping style, more studies are needed. Regarding the relationship of the FST with ALB, the available data support a low or null relationship (Andreatini and Bacellar, 1999; Naudon and Jay, 2005; do-Rego et al., 2006; unpublished data). To our knowledge only one paper has studied the consistency of immobility in the FST, using the classical pre-test/test procedure and comparing the immobility in the tests performed either 2 or 4 weeks apart (Drugan et al., 1989) and the correlation was reasonably good (*r* = 0.72 and 0.63, respectively).

We need more studies on test–retest consistency in tests of both ALB and depression-like behavior so far as this consistency is critical for proper assignation of each subject to a particular group, especially when only two groups are formed in function of the median. This problem is partially overcome if we assigned subjects to at least three groups (lowest, intermediate, and highest thirds) and all of them are further studied. This allows us to compare the two extreme groups, thus reducing the probability of incorrectly assigning animals to groups. Moreover, the intermediate group can give us information about the logic of the results obtained as we expect this group being in between the other two in any parameter of interest.

A well-known example of the use of non-genetically selected animals to study individual differences has been the characterization of the high responder (HR) and low responder (LR) phenotypes. This classification was based on their activity (high versus low, respectively) during prolonged exposure (up to 2h) in a novel environment. It was initially reported that activity of animals in a particular novel environment (a circular corridor) was related to the initial acquisition of amphetamine self-administration, with HR rats acquiring this behavior faster and more strongly (Piazza et al., 1989). Later it was observed a higher or more prolonged corticosterone response to stress in HR as compared to LR rats that may affect behavioral responsiveness to psychostimulants (Piazza et al., 1991). The rationale for focusing on corticosterone was the strong involvement of the hypothalamic–pituitary–adrenal (HPA) axis in stress and in several aspects of the response to addictive drugs (Piazza and Le Moal, 1998; Koob and Kreek, 2007). The activation of the HPA axis is a prototypical response to both systemic (i.e., hemorrhage) and emotional stressors (i.e., predator odor exposure) in all vertebrates. Systemic and emotional stressors are differentially processed within the brain but signals converge at the paraventricular nucleus of the hypothalamus (PVN), the key central controller of the HPA axis. The release of corticotrophin-releasing hormone (CRH) and other secretagogues formed in the PVN into the pituitary portal blood is the primary event leading to the activation of synthesis and release of adrenocorticotropic hormone (ACTH) in the anterior pituitary. This hormone controls the synthesis and release of glucocorticoids (cortisol in humans and most mammals, corticosterone in rodents) that exert numerous peripheral and brain effects important for the behavioral, physiological, and pathological consequences of stress. Another important characteristic of the activation of the HPA axis is that plasma levels of ACTH and corticosterone is consistently related to the intensity of stressors and can be therefore used as a biomarker of stress (Armario et al., 2012).

Other authors further confirmed, using a rectangular open-field, that HR rats showed higher HPA activity at both peripheral and central levels, including enhanced CRH gene expression in the PVN (Kabbaj et al., 2000). However, HR–LR rats also differed in anxiety. This is an important problem when we are trying to find biological correlates of specific behavioral traits and gives support to the importance of a more extensive behavioral characterization of the animals in these types of studies.

In order to know whether the differential corticosterone response was related to either novelty-seeking or anxiety trait, we exposed the rats to both a circular corridor and the EPM, demonstrating that activity in the circular corridor and time spent in the open arms of the EPM were completely independent variables. This allowed us to classify animals in function of novelty-seeking (HR–LR) or anxiety (HA–LA). We reported that HR showed higher HPA response to stressors than LR rats, confirming previous reports, whereas no differences were found between HA and LA rats (Márquez et al., 2006). The latter results appear to indicate that HPA responsiveness to stress is not a biomarker of anxiety and this conclusion is supported by other data presented in this review.

### CHARACTERIZING AFFECTED AND NON-AFFECTED INDIVIDUALS AFTER STRESS

The existence of individual or strain differences in susceptibility to develop hyper-anxiety or depression-like behavior under normal conditions or after stress is a good opportunity to search for the behavioral and neurobiological correlates of vulnerability. It is common to find controversial results in the literature, but in most cases not appropriate attention is given to the origin of the controversies. In addition to living conditions/facilities and procedural differences, a possible source of discrepancies is the genetic differences among currently used outbred mice and rat strains. This is a problem difficult to solve, but if there are consistent discrepancies among particular labs regarding the consequences of certain stress models, attention should be paid to the possible differences in the strain of animals they are using. It is more likely that discrepancies can appear when using inbred rat or mice strains, which nevertheless offer unique experimental possibilities (see later).

The usefulness of classifying the animals in function of the impact of stress and further exploring possible correlates is exemplified by the work done by Cohen’s lab with putative animal models of post-traumatic stress disorder (PTSD). They demonstrated in rats that a brief exposure to cat urine odor can trigger long-lasting (weeks) increases in ALB as measured by the EPM and the ASR. They classified the animals in function of the degree of alterations in ALB caused by odor exposure in well-adapted and mal-adapted (Cohen et al., 2003, 2005) for further exploring therapeutic strategies (Matar et al., 2006) or neurobiological changes associated to the differential response to cat odor (Kozlovsky et al., 2007). In some cases an additional intermediately affected group was also studied (Kozlovsky et al., 2007). This approach is very useful for the characterization of biological factors involved in vulnerability or resilience, but we cannot know whether differences were present before exposure to the triggering situation (i.e., stress) or they developed as a consequence of such an exposure. Transversal human studies have the same problem that can be solved with longitudinal studies. In animals, this problem can be overcome measuring certain behavioral and biological variables before and after the triggering situation. For instance, it has been reported in rats that ASR before exposure to a putative model of PTSD (one session of inescapable shock followed by weekly reminders) predicts the stress-induced enhancement of the ASR, which was only observed in those rats assigned to the top third group before exposure to stress (Rasmussen et al., 2008). Although these types of results need replication from different labs, it is a reasonable approach that parallels longitudinal studies in humans.

In the last decades, an animal model of chronic stress that uses chronic irregular exposure to a combination of different types of stressors over a period of one to several weeks has attracted considerable attention. This model of chronic stress was developed by Katz et al. (1981). The basic idea is that this stress model may be closer to human situations in which the type of stressors encountered daily as well as the time when they appear have a high degree of unpredictability and uncontrollability. The terms chronic unpredictable or chronic variable stress (CUS or CVS) have been used to refer to this model, but the term chronic mild stress (CMS), popularized by Willner’s laboratory in a series of seminal papers, starting in 1990 (Willner, 2005), is frequently used. The interest for CUS mainly derived from the reduction of sucrose consumption typically observed after 4 weeks of exposure to the stressors. As rodents like sweets, the reduction of sucrose intake is considered as a sign of anhedonia, a cue symptom of depression, which is corrected by antidepressants. It is frequently reported that such procedure enhances anxiety- and depression-like behavior, the latter reflected in anhedonia (i.e., reduced sucrose consumption) and passive coping strategies (i.e., increased immobility in the FST or the TST) (Willner, 2005; Hill et al., 2012).

Despite its extensive use, the CUS model has still important concerns. One is that the actual contribution of each particular stressor is not known, and it is difficult to predict the consequences of the different protocols used and which ones are the most appropriate. Another is related to the precise protocols used to evaluate sucrose preference (i.e., with or without prior food or water deprivation) and the extent to which changes in sucrose intake are an index of anhedonia. As chronic stress can reduce food intake, thus inducing a certain degree of anorexia, it is questionable the use of sucrose, which has caloric properties, to evaluate the purely hedonic properties of sweet solutions. This caveat is supported by a study in rat that observed reduction of sucrose but not saccharin consumption after CUS (Gronli et al., 2005). Regarding CUS-induced changes in anxiety, there are discrepant results, with absence of effects or even reduced anxiety in some cases (i.e., D’Aquila et al., 1994; Harris et al., 1998; Vyas and Chattarji, 2004; Mitra et al., 2005; Matuszewich et al., 2007; Kompagne et al., 2008).

It is likely that the origin of the discrepancies is at least in part due to genetically or environmentally determined differences in susceptibility among the different animals used. For instance, two reports have demonstrated the importance of individual differences by comparing two different outbred rat strains (Nielsen et al., 2000; Bekris et al., 2005). Two other studies have followed the approach of classifying animals in function of the impact of CUS, an approach that seems very promising with this particular model. Li et al. (2010) classified Wistar rats in anhedonic and non-anhedonic (reduction or not of sucrose preference) after CUS and observed also differences in other variables including behavior in novel environments and in the FST. Overall, CUS exposure resulted in reduced ALB in the open-field and EPM, but this anxiolytic effect was restricted to non-anhedonic (stress resistant) rats. These results nicely illustrate that the impact of CUS may be opposite if we are working with vulnerable versus resilience populations of animals. Using a similar criterion, Christensen et al. (2011) classified rats exposed to CUS as vulnerable or resistant and studied the differential gene expression in granular cells of the ventral dentate gyrus (taken by laser micro-dissection). More systematic studies using this approach are needed with CUS, a model of depression that is gaining acceptance among researchers and it is very good to explore individual differences in vulnerability to stress.

### GENETIC POLYMORPHISMS

In contrast to human studies, the impact of polymorphisms in animals is still in its infancy. However, some interesting results have been obtained regarding anxiety and depression. For instance, a rare single nucleotide polymorphisms (SNP) in the gene coding for vasopressin has been found in a normal population of Wistar rats that is more frequently present is those animals characterized by high ALB after genetic selection (Murgatroyd et al., 2004). This SNP in the regulatory region of the vasopressin gene resulted in enhanced vasopressin gene transcription in the PVN that appears to be strongly associated to the HA trait. Similarly, variations in the promoter region of a gene involved in the regulation of circadian rhythms (Per3) have also been associated to anxiety in mice (Wang et al., 2012). Two inbred rat strains that markedly differ in ALB, Lewis and spontaneously hypertensive rats (SHR), showed a SNP in the 3′-untranslated region of the α-synuclein gene, which codes for a (mainly) presynaptic protein associated to several brain diseases (Chiavegatto et al., 2009). This SNP results in enhanced expression of α-synuclein in the hippocampus and is associated with the enhanced anxiety of Lewis rats. SNP affecting depression-like behavior has also been reported. Thus, a C1473G polymorphism in the mouse tryptophan hydroxylase 2 gene (which code for the enzyme responsible for the brain synthesis of serotonin) is observed in several inbred laboratory mice but not in wild mice (Osipova et al., 2010) and those strains homozygotes for the G allele are characterized by high levels of inter-male aggression and immobility in the FST (Osipova et al., 2009). These types of studies are likely to contribute to a better comparison of animal and human studies.

## ANIMALS GENETICALLY SELECTED FOR ANXIETY

There are several well-characterized examples of animals genetically selected for ALB. The outbred Roman high avoidance (RHA) and Roman low avoidance (RLA) rats were obtained by genetic selection on the basis of their performance in a two-way active avoidance task (see Steimer and Driscoll, 2003). It was later found that the two lines differed not only in active avoidance, but also in terms of emotionality, the RLA rats being more emotional than RHA rats. The lines differ in some tests of anxiety more markedly than in others, being particularly relevant the inconsistencies regarding the EPM. A process of inbreeding has been carried out to obtain RHA and RLA strains that has essentially maintained the behavioral differences (Escorihuela et al., 1999). In Landgraf’s lab it has been obtained genetically selected lines of HA- and LA-related behavior (HAB, LAB) from Wistar rats and CD1 mice on the basis of the time spent in the open arms of the EPM (Liebsch et al., 1998b; Kromer et al., 2005). Similarly, genetic selection has allowed obtaining several lines of rats and mice showing depression-like behavior, including Flinders sensitive rats, congenitally learned-helplessness rats and H/Rouen mice (El Yacoubi and Vaugeois, 2007). Nevertheless, because the present review aims to identify strategies and problems associated with the characterization of individual differences, we will focus only on a few representative examples.

When we want to obtain genetically selected animals we use certain parameters to select the animals that are directly related to the problem of interest. The adequacy of the genetic process is evaluated measuring such behavior in each generation and the selection of the extremes in each generation. The selection process can result in two genetically heterogeneous outbred lines or in two genetically homogeneous inbred strains. We can thus eventually obtain stable lines/strains differences in the measure(s) of interest that are maintained across generations. If we are interested in selecting animals for ALB we can expose the animals to the EPM and choose a particular variable (i.e., the time spent in the open arms) to select the extremes of the population and mate males and females having the same phenotype to obtain two lines markedly differing in intensity. This has been the case of HAB–LAB rats (Liebsch et al., 1998b). The authors demonstrated that the two lines showed clear differences not only in the EPM but also in the D–L test (Henniger et al., 2000), what gives support to actual differences in ALB. In contrast, the ASR, which is considered to be positively related to anxiety, was lower in HAB as compared to LAB rats under normal conditions and after sensitization by prior exposure to footshock (Yilmazer-Hanke et al., 2004). Interestingly, factorial analysis revealed a higher contribution of anxiety and a lower contribution of activity to explain behavioral variability in HAB as compared with LAB rats during exposure to the EPM (Ohl et al., 2001).

On the other hand, HAB and LAB rats also appear to differ in another important trait, coping behavior, which identify whether animals are prone to develop passive or active strategies when facing novel aversive situations. During exposure to forced swim LAB rats show higher levels of active (struggling) behavior and lower levels of immobility than HAB rats (Liebsch et al., 1998b). These results obtained in HAB–LAB rats can be explained in two ways: (a) anxiety may markedly influence coping behavior, or (b) random genetic selection of genes resulted in parallel selection of genes influencing coping behavior. Factorial analysis can help to choose between the two hypotheses. As struggling behavior in the FST loaded on a different factor than EPM open arm entries and time spent in light in the L–D test (Salomé et al., 2002), it appears that the hypothesis of random selection of genes is more plausible. However, it is intriguing that a negative relationship between anxiety or high emotionality and active behavior in the FST has been repeatedly reported in genetically selected rat lines/strains (Abel, 1991; Paré and Redei, 1993; Piras et al., 2010), whereas no relationship appears to exist between classical anxiety measures and forced swim behavior in normal populations of rats and mice (earlier commented).

The above results illustrate some of the critical issues we are dealing with when selecting animals on the basis of a specific criterion for ALB. If animals also differ in other tests presumably related to the construct of anxiety (i.e., D–L, ASR), then we are more confident that the lines/strains really differ in anxiety. On the contrary, if no differences are observed in other tests, we have to be more cautious and assume that the selected lines only differ in certain aspects of anxiety.

In searching for the neurobiology of behavioral traits, it is common to study whether two selected lines also differ with respect to some biological variables of interest based on specific hypotheses about such a relationship. For instance, attention has been devoted to the putative relationship between anxiety and the activity of the HPA axis usually comparing a pair of lines differing in anxiety. Unfortunately, such studies can lead to spurious and confusing results due to the already discussed random selection of genes specifically involved in the control of anxiety or in the control of the HPA axis. For instance, there is a reasonable degree of accordance in that RLA (HA) lines/strains showed an enhanced HPA responsiveness to stress as compared to RHA (LA) lines/strains. This is true particularly regarding emotional or predominantly emotional stressors but not systemic stressors (i.e., Gentsch et al., 1982; Carrasco et al., 2008). However, the comparative HPA response of HAB–LAB rats to stressors showed different results depending on the particular stressor used (Liebsch et al., 1998a; Landgraf et al., 1999; Frank et al., 2006). The lack of consistent relationship between anxiety and HPA responsiveness is also supported by data in a non-genetically selected population of Sprague-Dawley rats where ACTH and corticosterone responses to novel environment did not differ in HA or LA rats (Márquez et al., 2006). In fact, available data are, not unexpectedly, deceptive and the overall analysis strongly indicates that there is no relationship between trait anxiety and the activity of the HPA axis either in humans or laboratory rodents (Courvoisier et al., 1996; Ramos and Mormède, 1998; Solberg et al., 2003; Armario and Nadal, 2013).

There are several main lessons from those studies. First, if we want to establish a relationship between the HPA axis and anxiety (or other behavioral trait) we need to evaluate the HPA response: (a) to emotional rather than systemic stressors; (b) to different types of emotional stressors; and (c) in different pairs of lines/strains selected for similar criterion. If this approach is not followed and our conclusions are based on less complete experimental designs we need to be aware of the limitations of our study and the possibility to obtain spurious relationships. More importantly, if such a relationship is accepted by other researchers is a matter of fact or as working hypotheses in order to further explore its precise neurobiological substrate, we will invest considerable effort in the wrong way. In addition, if we search for similar relationship in other lines apparently selected for the same or similar criterion the results should be necessarily inconsistent, introducing a high level of noise in the literature.

In sum, it is important to realize that each particular test can capture only certain aspects of anxiety and can be influenced by factors other than anxiety. This is particularly important when we want to know whether classification of a normal or genetically selected population of animals in function of a particular variable can also result in changes other behavioral aspects presumably influenced by anxiety. It can be expected that the relationship with other behavioral aspects is strongly influenced by the criterion used for the selection of anxiety. The same applies to the possible relationship of anxiety with any biological parameter such as the HPA axis. There are no easy solutions to these problems, but one possible strategy is to include more than one test in the selection criterion. A complementary one during genetic selection is to simultaneously select several different pairs of lines (2 or 3) from the same original population and the same criterion and then, once the different pairs were stable, to simultaneously study all lines when trying to relate the chosen trait with other behavioral or physiological characteristics. If we introduce more than one test to characterize any behavioral trait or several different stressors for the evaluation of the HPA axis, we can be more confident about the significance of the findings. We are aware that this is expensive and time consuming, but could be feasible with the joint effort of various labs and can contribute to clarify important controversies.

## EXPLOITING ALREADY AVAILABLE STRAIN DIFFERENCES

Rather than selecting animals for a specific criterion we can take advantage of the use of the considerable number of already available genetically selected lines/strains of rats and mice. Inbred and recombinant inbred strains have been an excellent genetic tool (Nguyen and Gerlai, 2002) and considerable attention has been paid to baseline or stress-induced anxiety in available rats or mice inbred strains. The problem of random genetic selection can affect any genetically selected animal, but it is expected to be worst with inbred than outbred strains as genetic variability is completely reduced in the formers. If specific alleles influencing any specific (physiological or behavioral) trait are randomly fixed in all subjects, the distortion is likely to be greater than in genetically selected outbred populations. As most genetically modified mice are obtained from particular inbred strains, it is not surprising to find important phenotypic differences after genetic modifications depending on the genetic background of mutant animals. Thus, null mutation of the serotonin transporter was found to increase anxiety in the C57BL/6J background, but not in the 129P1/Rej background (Holmes et al., 2003). The genetic background has influence on some particular tests for anxiety as deletion of the pro-enkephalin gene increased anxiety as evaluated with the L–D test and the ASR in the C57BL/6J background, whereas in the DBA/2J background the deletion increased anxiety in the zero maze and the social interaction test (Bilkei-Gorzo et al., 2004).

There are numerous reports describing baseline differences in ALB and depression-like behavior as well as differences in the responsiveness to anxiolytics and antidepressants among commercially available outbred and inbred rodent strains. Before discussing some available data, it is important to take into account the general problem of possible differences in pharmacokinetics. As a matter of fact, only some studies comparing group or strain differences in the response to psychotropic drugs presented data about pharmacokinetics. If such data are not presented, it is critical to compare at least two functionally unrelated responses to the drug (Lahmame and Armario, 1996; Belzung, 2001). If differences in sensitivity to the drugs among the groups are similar, independently of the function studied, a major contribution of pharmacokinetics should be expected. On the contrary function-dependent differences may be suggestive of pharmacodynamics differences. In addition to pharmacokinetics, there are other reasons for this differential response that has been previously discussed regarding anxiolytics (Belzung, 2001) and they will be only briefly summarized. One is that drugs would be more effective in those subjects or strains showing higher baseline levels of ALB or depression-like behavior. Although this hypothesis is frequently supported by studies on environmentally induced changes in behavior, this does not always applies to an important number of examples with available outbred or inbred rodent strains (see Belzung, 2001 for review). An additional explanation is that pharmacodynamics differences may exist related to the functional activity of neurotransmitters and receptors or neural circuits critically involved in the control of ALB. This differential response could be the fundamentals for the elucidation of underlying alterations in neurotransmission or circuits.

The above consideration can also be applied to depression-like behavior. Rat and mice strain differences in responsiveness to the tricyclic antidepressant imipramine were firstly reported by Porsolt et al. (1978) in the FST. In mice, strain differences in the response to several antidepressants using the FST or TST were also reported (van der Heyden et al., 1987; Lucki et al., 2001). Comparison of different inbred rats strains revealed striking differences in the response to several antidepressants that are not related to baseline differences in forced swimming behavior or drug pharmacokinetics (Lahmame and Armario, 1996; Lahmame et al., 1997; López-Rubalcava and Lucki, 2000). Further studies have allowed associating altered responsiveness to antidepressants with particular biological characteristics. For instance, the deficient response of DBA/2J and BALB/c mice to the selective serotonin reuptake inhibitor citalopram appears to be dependent on the integrity of the serotoninergic system and is related to impaired serotoninergic synthesis (Cervo et al., 2005).

The CUS procedure is also being used to approach individual or strain differences in susceptibility to ALB and depression-like behavior. Crusio’s and Belzung’s labs have obtained interesting data comparing CBA/H, C57BL/6, and DBA/2 inbred mice strains. They found that CBA/H and C57BL/6, but not DBA/2 mice, showed decreased sucrose consumption after CUS (Pothion et al., 2004). Further, it was demonstrated that the effect of CUS was dependent on sex, strain, and the particular type of task used to evaluate anxiety and depression-like behaviors (Mineur et al., 2006). Similarly, whereas CUS reduced neurogenesis in the subgranular (dentate gyrus) and subventricular zones and impaired hippocampus-dependent but not hippocampus-independent learning tasks, there was not clear relationship between changes in neurogenesis and changes in behavior across strains and sexes (Mineur et al., 2007). Another study not only demonstrated that BALB/cByJ mice are more emotional than C57BL/6, but that classification of mice from both strains into high or low emotional on the basis of their response to the EPM and free exploratory tests resulted in higher impact of CUS in the more emotional (Ducottet and Belzung, 2004).

It is of note that an important degree of individual differences are usually observed in inbred animals that are likely to be due to the influence of environmental factors through epigenetic processes (Jakovcevski et al., 2008). In this regard, there are important differences among the breeding procedures used in the various provider centers and this information should be made available to researchers. For instance, litter size, sex ratio, disturbances of the litter during weaning, individual versus communal nesting, age of weaning, number of animals per cage during the post-weaning period or at adulthood are scarcely reported factors. Attention should also be paid to the possibility of genetic differences between supposedly inbred strains. The Wistar Kyoto (WKY) is an inbred strain of rats that has been reported to show depression-like behavior in the FST (Paré and Redei, 1993; Martí and Armario, 1996), but the response to antidepressants seemed inconsistent. Quite interestingly, Will et al. (2003) obtained two genetically derived sub-strains of WKY rats that showed a clear differential response to some antidepressants, explaining prior controversial results. The origin of this genetic variability is still unknown. The above mentioned factors can explain the important discrepancies between laboratories in the FST behavior of particular outbred and inbred mice either in drug-free conditions or in response to antidepressants (Lucki et al., 2001; Ventura et al., 2002; David et al., 2003; Dulawa et al., 2004; Cervo et al., 2005; Guzzetti et al., 2008; Sugimoto et al., 2008).

## GENETIC MANIPULATION OF TARGETED GENES

More classical approaches have been genetically driven silencing or over-expression of a particular gene. Again, approaches strongly differ in humans and animals, thus making it more difficult to translate results from bench to clinic.

The introduction of genetically modified animals has represented an extraordinary advance in biomedicine, but there are important problems associated with this approach, including the possible developmental consequences of the altered expression of a gene in the embryo or the lack of tissue/cell specificity. Important concerns have been more recently overcome by the use of conditional and more tissue selective mutations. Since this topic has been extensively discussed, we would like to focus only on a less discussed problem. Gene polymorphisms within a normal population of animals can modify (increasing or decreasing) the expression of this gene or its function, whereas genetic modifications can completely block expression or cause a non-physiological over-expression. It is obvious that these non-natural modifications are far from those usually observed in natural populations. Therefore, any observed consequence of such genetic manipulation can be viewed with great caution when thinking about the actual impact of more modest natural alterations of gene expression or function. Over-estimation of the impact of a particular gene can detract attention from other genes and create expectancies that are not further supported by more real data. This problem is in great part solved by the inclusion in the studies of heterozygote animals. Thus, neurochemical characteristics of heterozygote 5-HTT^+^^/^^-^ mice are close to the presence of the two short alleles of the serotonin transporter (5-HTT) in humans (Murphy et al., 2001), which is associated with trait anxiety (Lesch et al., 1996). Under minimal stressful conditions, heterozygote mice behave as wild-type in several different tests for anxiety, whereas homozygote mice showed clear signs of enhanced anxiety (Holmes et al., 2003). Interestingly, heterozygote mice appear to be more sensitive to the negative impact of early life experience on anxiety (Carola et al., 2008), suggesting enhanced vulnerability to stress similar to that observed in humans carrying the short allele of the 5-HTT gene (Caspi et al., 2003). Although some results are sometimes difficult to replicate, these data indicate that we can design strategies in animal research closer to human studies. Considering the polygenic nature of human pathologies, particularly important in this regard is the possibility of studying the interactions between different genes in animal models (Murphy et al., 2003).

A less explored approach with high translational value is the introduction in mice of genes having natural human mutations associated to vulnerability or pathologies. A most relevant case is that of the val66met polymorphism of the brain-derived neurotrophic factor (BDNF) neurotrophin, which impairs activity-dependent BDNF secretion (Egan et al., 2003) and results in enhanced stress-induced anxiety (Chen et al., 2006) and impaired fear extinction learning in both mice and humans (Soliman et al., 2010).

## SEX DIFFERENCES

The present review does not specifically address sex differences, but some key points will be discussed. Epidemiological studies in humans indicate that the prevalence of certain psychiatric disorders such as depression (dysthymia, major depression, atypical, and seasonal) and anxiety (generalized anxiety, post-traumatic stress, and panic) are clearly higher in females than males (Toufexis et al., 2006), although the precise contribution of biological, social and cultural factors is still unclear. In addition, some differences in the therapeutic response to drugs have also been reported (Marazziti et al., 2013) and there is evidence for sex differences in pharmacokinetics of antidepressants in humans, although their clinical relevance is still under debate (Kokras et al., 2011). For all the above reasons, there is now a renewed interest for the study of sex differences in animal models of psychopathologies.

Sex differences in ALB and depression-like behavior in rats and mice have sometimes been reported, but the bulk of results did not favor the hypothesis that major differences exist. Activity in novel environments (including the EPM) is consistently higher in female rats and this can explain the greater number of open and closed arm entries in the EPM (Lucion et al., 1996; Fernandes et al., 1999; Gulinello and Smith, 2003; Simpson et al., 2012). However, most papers failed to demonstrate specific differences in anxiety when taking into account the time spent in the open arms and tests other than the EPM (Johnston and File, 1991; Lucion et al., 1996; Stock et al., 2000; Gulinello and Smith, 2003; Braun et al., 2011). It is possible that female rats display lower anxiety restricted to some specific ages (Imhof et al., 1993) and to the pro-estrous phase of the estrous cycle (Mora et al., 1996; Frye et al., 2000). Interestingly, direct comparison of Sprague-Dawley and FSLs has demonstrated no sex differences in time spent in the open arms in the former strain but clearly greater levels in females as compared with males from the FSL (Kokras et al., 2011). This suggests that some genetic selection processes could differentially alter anxiety in the two sexes. In mice, results showed no sex differences (Rodgers and Cole, 1993). The anxiolytic effects of diazepam in the EPM has been found to be similar in either intact or gonadectomized rats of both sexes (Stock et al., 2000; Wilson et al., 2004). In contrast, in another study in which lower baseline anxiety was observed in females than males, the former did not respond to two different doses of diazepam whereas males did (Simpson et al., 2012). Whether the latter results are suggestive of a lower sensitivity of females to anxiolytics or can be explained by pre-drug differences remains unclear. It is important to realize that sex differences are the results of the evolutionary history of each species and that perhaps rats and mice are not the appropriate species to model sex differences in anxiety and emotional behavior humans (Donner and Lowry, 2013).

It is unclear whether or not female rodents show more active coping behavior in the FST. It was initially reported that females showed lower levels of immobility than males and that immobility was not affected by the estrous cycle (Alonso et al., 1991). These results were further confirmed (Barros and Ferigolo, 1998; Simpson et al., 2012), but other reports have shown less clear effects (Kokras et al., 2009, 2011; Morrish et al., 2009) or even greater immobility in females (Dalla et al., 2008; Kokras et al., 2009). Null or minor sex differences are consistently observed in mice in the FST and TST (David et al., 2001; Caldarone et al., 2003; Jones and Lucki, 2005; Steiner et al., 2008; Andreasen and Redrobe, 2009). Inconsistent results have also been reported regarding the action of antidepressants, with less sensitivity to chronic fluoxetine or citalopram in females (Lifschytz et al., 2006; Gunther et al., 2011) or similar response to desipramine, clomipramine, or fluoxetine (Monteggia et al., 2007; Jacobsen et al., 2008; Dalla et al., 2010). In one study, no differences appeared to exist in mice between sexes in the effects of amitriptyline on the TST and FST, despite a differential response in the learned-helplessness paradigm (Caldarone et al., 2003), another putative model of depression. Nevertheless, the neurobiological substrate of behavior in the FST appears to differ in male and female mice as conditional knocking of the bdnf gene increased depression-like behavior in females but not males (Monteggia et al., 2007). Therefore, although the performance in some particular tests may be similar in males and females, it is likely that this can be achieved by different neurobiological mechanisms.

## CONCLUSION

Development of appropriate animal models to induce and test behavioral changes reminiscent of those observed in human psychiatric disorders as well as to identify factors of differential susceptibility to develop such disorders is still a great challenge. This is due in part to the complexity of the problems with are dealing with, but also to naïve approaches, which pay not enough attention to well-described methodological concerns. Results obtained in genetically selected animals (outbred or inbred) can be viewed with caution and it is recommended to compare several different lines or strains to reduce the probability of obtaining spurious relationships when searching for the biological substrate. A combination of genetic selection and experimental manipulation of target molecules is critical to reveal causality. Moreover, the polygenetic nature of psychiatric diseases makes it likely that only animal having genetic changes in more than one target gene could approach to human disease.

Some of the approaches used in animals are not possible in humans, but we can develop animal models as close as possible to human studies. This includes the study of the consequences of natural polymorphisms in the same genes in animal and humans populations, the introduction in mice of human genes associated with certain pathologies by genetic engineering, and the use of heterozygotes in genetically modified animals. Parallel approaches in humans and animals, when possible, can help to uncover methodological problems and to advance faster. The characterization of the biological bases of individual differences is likely to be one of the great challenges of biomedicine in the next future.

## Conflict of Interest Statement

The authors declare that the research was conducted in the absence of any commercial or financial relationships that could be construed as a potential conflict of interest.
